# Cerebral amyloid angiopathy related inflammation: A little known but not to be underestimated disease

**DOI:** 10.1016/j.radcr.2021.05.080

**Published:** 2021-07-03

**Authors:** Daniela Grasso, Giulia Castorani, Carmela Borreggine, Annalisa Simeone, Roberto De Blasi

**Affiliations:** a IRCCS Ospedale Casa Sollievo della Sofferenza, Department of Diagnostic Imaging, San Giovanni Rotondo, Italy; b University of Foggia Postgraduate Medical School, Diagnostic Imaging, Foggia, Italy; c Department of Diagnostic Imaging, Tricase, Italy

**Keywords:** Cerebral amyloid angiopathy, Inflammation, MR, Susceptibility weighted imaging, Microbleeds

## Abstract

Cerebral amyloid angiopathy related inflammation (CAA-ri) is a rare encephalopathy resulting from perivascular inflammation after β-βamyloid (A) deposition in cerebral vessels leading to progressive dementia, focal neurological signs, seizures and intracerebral hemorrhages. This condition is characterized on magnetic resonance imaging (MRI) by patchy or confluent T2/fluid attenuation inversion recovery (FLAIR) hyperintensities in the cortex and subcortical white matter located mainly in the same areas of pre-existing multiple microhemorrhages. In this report of 2 cases of “probable” CAA-ri women aged 71 and 68, we propose a review on the pathophysiological, clinical, radiological, therapeutic and prognostic aspects of this little-known and poor outcome condition.

Even though an apparently favorable initial evolution after steroid and/or immunosuppressive treatment, CAA-ri course is unpredictable and often associated with low survival rates. We suggest the importance of timely and proper clinico-radiological evaluation in suspected CAA-ri cases, in order to start an appropriate treatment even without the brain biopsy.

## Introduction

Cerebral amyloid angiopathy related inflammation (CAA-ri) represents an unusual and life- threatening manifestation of Cerebral amyloid angiopathy (CAA), a frequent age-related encephalopathy, with an unfavorable outcome in the majority of the patients. It is subsequent to β amyloid storage in the media and adventitia of the small and medium sized arteries and capillaries of the cerebral cortex and the leptomeninges [1], [2], [3] that induces severe vascular dysfunctions with increased risk of cortical-subcortical or lobar hemorrhages [4], [5], [6].

Radiological findings and clinical data play a key role in the diagnosis of this rare condition according to Chung criteria [7], [8], [9], even if the definite diagnosis of CAA-ri requires brain biopsy [10], [11], [12]. There are no specific diagnostic lab tests or serologies [13] although an increasing number of studies has recently investigated the contribution of autoantibodies against amyloid β in the CSF of CAA-ri subject [14], [15], [16], [17], [18]. There is no sex predilection of CAA-ri but the onset of symptoms with acute or subacute manifestations is earlier than CAA and includes seizures, headache, behavioral changes, cognitive decline and focal deficits [19], [20], [21], [22], [23].

Despite an apparently favorable initial evolution after a prompt empirical immunosuppressive therapy [24], CAA-ri is characterized by a poor prognosis attributed to different factors such as CAA-relapses, intracerebral hemorrhages (ICH) and infectious or vascular complications [25], [26].

We report two cases of probable CAA-ri supported by clinical and radiological evidences and their serious complications.

## Case report 1

A 71 years old woman presented to our hospital with dysarthria and impaired ambulation with a clinical history of progressive worsening of left hemibody myoclonus, facial nerve dysfunction, and mild diffuse hypertonia.

The patient has made some admissions in other hospitals with a progressive worsening of clinical and behavioral symptoms becoming noncompliant, in soporous state, hostile, and disoriented.

On admission laboratory tests disclosed hypogammaglobulinemia and severe lymphocytopenia.

Electroencephalogram (EEG) was normal. Hyperactive responses using deep tendon reflexes tests (DTR) was observed.

The patient was treated with bolus administration of steroids and with cyclophosphamide and azathioprine.

A brain MRI study revealed cortical-subcortical abnormal hyperintensities areas on FLAIR images in both cerebral hemispheres. SWI imaging demonstrated several punctate hypointensities, referred to cortical ferromagnetic storages, in particular hemosiderin within the above areas not sparing the subcortical U-fibers.

**Fig. 1:**
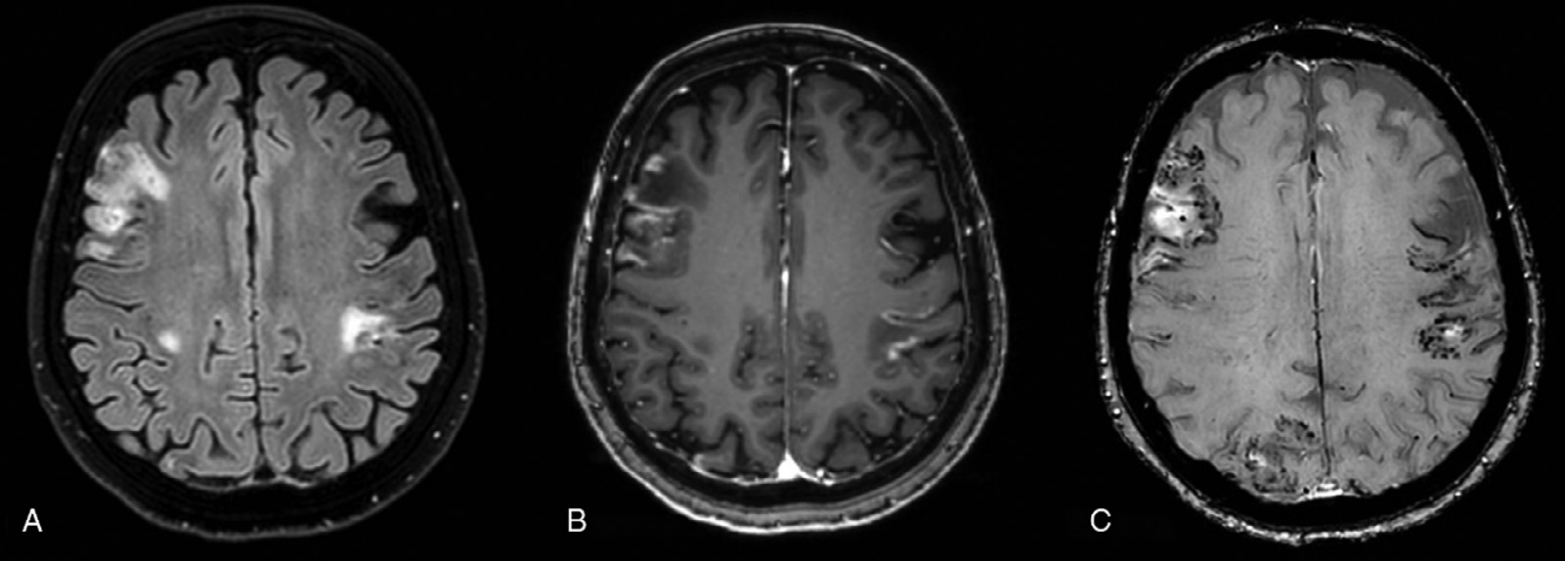
CASE 1 (A) FLAIR sequence showed abnormal cortical-subcortical lobar hyperintensities in both cerebral hemispheres. (B) Postcontrast axial T1 images demonstrated marked gyral enhancement in the same areas. (C) Multiple and bilateral cortical–subcortical microhemorrhages were seen on postcontrast Susceptibility weighted images corresponding to FLAIR cortical-subcortical hyperintensities.

After the intravenous administration of Gadolinium, all of the mentioned lesions showed gyral enhancement on post-contrast T1 W images (Fig. 1)

Thereafter, the patient was transferred to hospice care with oral prednisone treatment until the exitus occurred few weeks later.

## Case report 2

A 68 years old woman was admitted in our hospital due to the sudden onset of paresthesia and spastic paraparesis of the left leg and sphincters’ dysfunction and with a history of tension headache, confusion, nausea and vomiting. She presented positive left Babinski sign, hyperactive responses of deep tendon reflexes, normal cognitive function and cranial nerves. Routine laboratory tests, serological tests (including the rheumatological ones) and CSF were unremarkable except for positive ANA (antinuclear antibody).

**Fig. 2:**
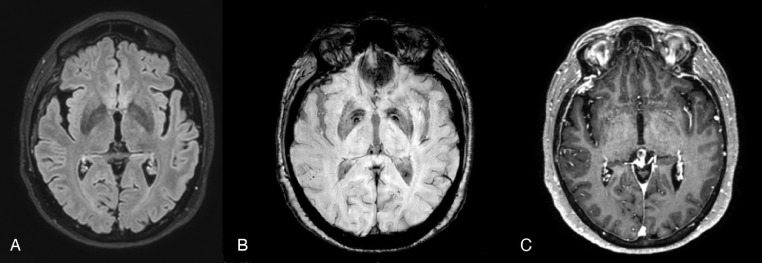
CASE 2 (A) FLAIR ax; (B) SWI ax; post contrast T1 MPRAGE ax. The first MRI study showed a little cluster of cortical-subcortical hypointense foci on Susceptibility weighted imaging (SWI) (B) sited in the right superior temporal gyrus and near the angular gyrus, without abnormal signal of the surrounding white matter on FLAIR images (A). No enhancement was seen (C).

She underwent contrast-enhanced magnetic resonance imaging (MRI) of the brain and spine, which revealed a little cluster of cortical-subcortical and peripherical hypointense foci on Susceptibility weighted imaging (SWI) sited in the right superior temporal gyrus and near the angular gyrus, probably due to micro-hemorrhages (cerebral microbleed-CMB), without abnormal signal of the surrounding white matter on T2W and FLAIR images. During hospitalization she was administered bolus steroids (Fig. 2).

Seven months later a follow up brain MRI disclosed a severe spreading of punctate microbleeds on SWI in cortical and cortical-subcortical areas of both hemispheres, in the brainstem and in the cerebellum. Some of CMBs tended to cluster, leading to macrohemorrhages, in both temporo-parietal region, superior frontal gyri, and cingulate gyri and in the right inferior cerebellar hemisphere.

**Fig. 3:**
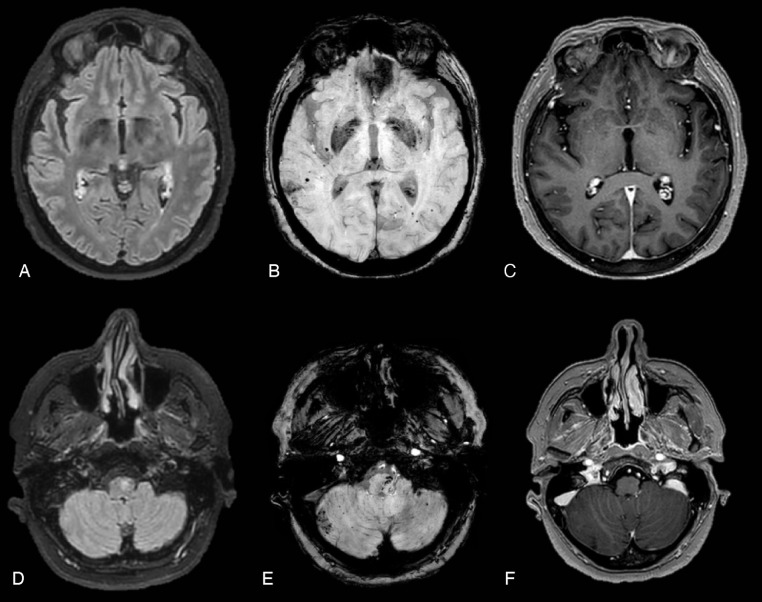
(A, D) FLAIR ax; (B; E) SWI ax; (C-F) post contrast T1 MPRAGE ax. Seven months later a brain MRI disclosed spreading of punctate microbleeds on SWI (B) in cortical and cortical-subcortical areas of both hemispheres, in the brainstem and in the cerebellum. Some of CMBs tended to cluster, leading to macrohaemorrhages, in both temporo-parietal region and in the right inferior cerebellar hemisphere. On FLAIR images (D) WM hyperintensity was observed in the left bulbar pyramid where a serpiginous hypointensity on SWI was detected. No enhancement was seen in supra and subtentorial regions.

Additionally, a blurred WM hyperintensity on long TR sequences was observed only in the left bulbar pyramid where a serpiginous hypointensity on SWI was detected (Fig. 3).

No further abnormal signal of the surrounding white matter was noticed neither diffusion restriction on ADC map.

According to the suspicion of an inflammatory or rheumatological disease, the patient received steroid bolus and Azathioprine**.**

**Fig. 4:**
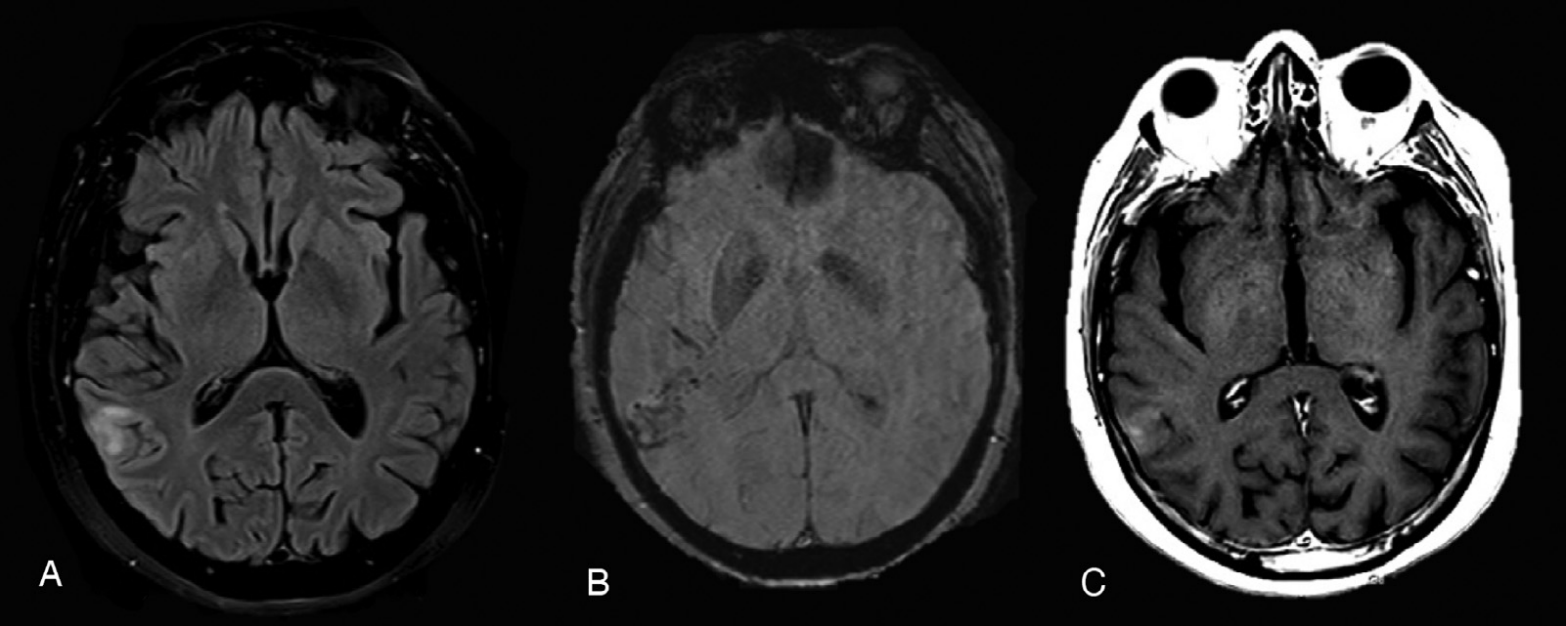
(A) FLAIR ax; (B) SWI ax; post contrast T1 SE ax. Two months later a follow-up MRI study showed the onset of a subcortical area of white matter hyperintensity on FLAIR images (A) in the right parieto-temporal region arising on previous haemorragic foci (B). After contrast administration, the exam showed blurred contrast-enhancement in the same involved WM area (C).

She represented 2 months later in a soporous state after a generalized tonic-clonic seizure. A brain MRI was performed and disclosed the onset of a subcortical area of white matter hyperintensity in the right parieto-temporal region arising on previous hemorrhagic foci, described in the past MRI. After contrast administration, T1-sequences showed blurred contrast-enhancement in the same involved WM area (Fig. 4).

The radiological and clinical course of the patients corroborated the past hypothesis of an inflammatory disease, suggesting CAA-ri, and therefore she was discharged with prescription of Cyclophosphamide, oral prednisone and imaging follow-up.

The patient underwent a new brain-MRI study one month later, showed the previous right parieto-temporal WM area of hyperintensity almost disappeared, as well as its contrast enhancement.

Conversely, a dramatic dissemination of new cerebral microbleeds appeared on the SWI in both hemispheres, basal ganglia, brainstem and cerebellum. The multiple CMBs had been increased in number and dimension leading to larger cerebral hemorrhages.

**Fig. 5:**
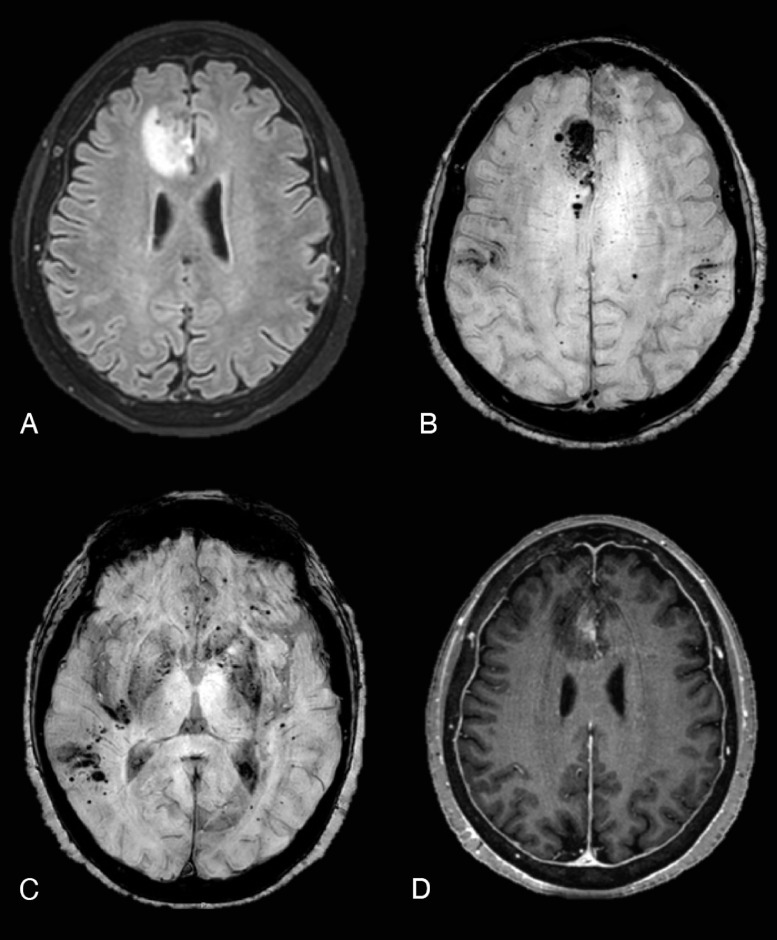
(A) FLAIR ax; (B) (C) SWI ax; (D) postcontrast T1 MPRAGE. One month later, the last MRI study showed a dramatic dissemination of new cerebral microbleeds appeared on the SWI in both hemispheres, basal ganglia, brainstem and cerebellum (B-C). FLAIR sequences (A) revealed the onset of a new area of vasogenic edema in the right superior frontal gyrus close to the pre-existing cluster of microbleeds detected in lasts controls. Dishomogeneous enhancement was seen in the same area on post contrast T1 MPRAGE (D).

In addition, T2-wi and FLAIR sequences revealed the onset of a new area of vasogenic edema in the right superior fontal gyrus close to the pre-existing cluster of microbleeds detected in lasts controls (Fig. 5).

The patient was given methylprednisolone pulse therapy for 3 days followed by oral prednisone and then she was discharged home on steroid therapy.

However, 3 months later, the subject was admitted again in our hospital owing to the occurrence of cyclophosphamide-induced jaundice and acute renal failure. Moreover, the CT revealed a portal and mesenteric vein thrombosis and subcutaneous Enoxaparin therapy was performed. Unfortunately, the patient's condition rapidly deteriorated in the following days till exitus.

## Discussion

Cerebral amyloid angiopathy (CAA) identifies a heterogeneous group of disorders caused by deposition of an β amyloidogenic protein in cortical, leptomeningeal and cerebellar vessels and rarely in the brainstem or basal ganglia [1]. It results from an error related to three steps: production of amyloid precursor proteins; processing of the precursor proteins to amyloid proteins; and aggregation of the amyloid proteins and fibril formation [2]. CAA arises mostly as a sporadic condition in the elderly or as hereditary familial form [3]. Actually, it embraces a recently recognized syndrome of reversible encephalopathy accompanied by perivascular inflammation or edema: CAA-ri.

The toxic effect of Aβ deposits activates a cascade of biochemical process leading to disruption of the cerebrovascular architecture promoting intracerebral micro and macro-hemorrhages (ICH), which are often associated to CAA-I [4], [5], [6]. MRI leads a central role in detecting these ICH on GRE T2 or susceptibility weighted (SWI) sequences as multiple cortical and cortical-subcortical hypointensities (microbleeds), selectively distributed within the pre-existing white matter lesions.

Typical radiological findings on MRI are asymmetrical reversible long TR sequences hyperintensities of the white matter associated to multiple lobar microbleeds, with or without leptomeningeal or parenchymal gadolinium enhancement. White matter lesions, probably due to vasogenic edema, generally figures as reversible asymmetrical patchy or confluent hyperintense areas on long TR sequences, sited in the subcortical white matter and less frequently the contiguous gray matter. Furthermore, slight leptomeningeal enhancement after contrast administration may be present or not [7].

All of the mentioned above radiological signs contribute to identify the “definite” or “probable” CAA-ri based on the Chung's analysis of 72 patients with CAA-ri (Table 1) [8,9]. In the present two cases, the combination of progressive neurologic symptoms with frequent relapses associated to typical radiological findings led to suspect “probable” CAA-ri. Unfortunately, brain biopsies, required for a definite diagnosis, were not performed.

**Table 1:** Proposed diagnostic criteria for cerebral amyloid angiopathy related inflammation (CAA-RI).

PROBABLE CAA-RI
All the following: 1. Acute or subacute onset of symptoms. 2. 40 years of age or older. 3. At least one of the following clinical features: headache, mental status or behavioural change, focal neurological signs and seizures. 4. MRI shows patchy or confluent T2 or fluid attenuation inversion recovery hyperintensity which is: a. usually asymmetric b. with or without mass effect c. with or without leptomeningeal or parenchymal enhancement. 5. Evidence of pre-existing CAA on susceptibility weighted MRI sequences: a. multiple cortical and subcortical haemorrhages or micro- haemorrhages and/or b. recent or past lobar hemorrhage 6. absence of neoplastic, infectious or other cause.
DEFINITE CAA-RI

All of the above plus histopathological confirmation with: 1. Perivascular, transmural and/or intramural inflammation 2. Amyloid deposition within vessels of affected area in the cortex and leptomeninges.

Unfortunately, brain biopsies were not performed. Even if the histopathological examination is required for a definite diagnosis, the Chung criteria represent an essential tool for early recognition of this disorder, avoiding unnecessary invasive procedures [9]. Histopathologically, CAA-ri is distinguished by vascular amyloid deposition and inflammatory infiltration by lymphocytes, eosinophils, and multinucleated giant cells, resembling both CAA and central nervous system vasculitis [10]. It could be divided into two subtypes: Aβ- related angiitis (ABRA) and inflammatory-CAA (ICAA). The first one is an angiodestructive process of vasculitic transmural infiltration. The second one is described as an inflammatory reaction around amyloid-laden vessels, without angiodestructive action [11]. Despite clinical and imaging data are not sufficient to clearly differentiate the two entities, Chu et al suggested that gadolinium enhancement on MRI is predictive of ABRA while ApoE 4/ 4 genotype of CAA-ri [12]. Inflammatory markers have a limited role and CSF examination contributes to exclude infections or malignancies like in our reported cases [13]. However, several authors recently discovered raised autoantibodies against amyloid β in the CSF of CAA-ri patients during acute symptoms that, importantly, progressive decrease after each steroid pulse and return to control levels in parallel with clinical and radiological remission [14], [15], [16]. Nevertheless, Piazza et al [15], observed a similar spontaneous decreased of antibodies concomitant the patients’ improvement even without the steroid's administration, and considered this trend strictly related to the CAA-ri immune pathogenetic mechanism and not only to the efficacy of therapies. Furthermore, the authors pointed out the close correlation between CAA-ri and the recently defined amyloid-related imaging abnormalities (ARIA) reported in Alzheimer's disease (AD) passive immunization therapies. The term ARIA was coined in 2010 by the Alzheimer's Association Research Roundtable Workgroup and classified vasogenic edema and/or sulcal effusion as ARIA-E and CMBs or other small areas of hemosiderosis as ARIA-H in the AD patients treated with amyloid-modifying therapies (such as Aβ monoclonal antibody bapineuzumab) [17]. Piazza et al. proposed CSF Aβ autoantibodies as novel biomarkers of both CAA-ri and ARIA speculating that CAA-ri could represent a human spontaneous model of the drug-induced ARIA in AD. Despite the underlying trigger for this antibody response is not yet known, ARIA and CAA-ri share a similar spectrum of MR abnormalities and clinical features. They both are associated with APOE e4 allele dose too [18]. Several clinical conditions were considered as differential diagnosis of CAA-ri: autoimmune encephalitis, acute disseminated encephalomyelitis, neurosarcoidosis, reversible posterior leukoencephalopathy syndrome, malignancy and infection [19]. CAA-I may simulate an acute stroke (ischemic and hemorrhagic) due to overlapping radiological imaging [20]. First of all, one of the main radiological clues in our report was the sudden onset of multiple cortical-subcortical intracerebral hemorrhages. PRES (posterior reversible encephalopathy syndrome) rarely manifests ICH and especially in advanced disease, arising in the previous areas of white matter edema which usually involves symmetrically the parieto-temporo-occipital lobes, while in CAA-ri ICH represents the primary cause of vasogenic edema [21]. More challenging is to differentiate the CAA-ri from primary CNS vasculitis (PCNSV). According to Salvarani et al., CAA-ri, especially the ABRA subtype, could represent a definable subset of PCNSV even if with less frequent cerebral infarction and more common gadolinium leptomeningeal enhancement and intracerebral hemorrhage [22]. Another recurrent cause of cerebral macro and microbleeds (CMBs) in adults is the chronic hypertensive microangiopathy commonly sited in basal ganglia, thalamus, brainstem and cerebellum, sparing cortical and subcortical areas despite the predominant lobar dissemination of our patients [23].

According to the highly predictive of CAA-ri imaging features, the immunosuppressive treatment was started in our patients, with high-dose parenteral corticosteroids followed by oral corticoids, associated with Cyclophosphamide or Azathioprine. As already reported in the literature [24], both patients rapidly improved after immunosuppression treatment. However, they dramatically worsened over the months due to different relapses, severe cerebral radiological deterioration and progressive hepatic, renal and vascular complications until exitus. Coulette et al. outlined the crucial impact of an early treatment of CAA-ri leading to less frequent relapses in patients than in the untreated ones [25], however our report outlined that long-term recurrences are frequent and life- threatening, especially due to infectious complications after immuno-suppressant treatment or progressive ICHs. In particular, like in one of our subjects, a venous thrombotic accident in a CAA-ri patient should be considered an insidious complication because of the risk and benefit ratio of the antithrombotic therapy, as indicated by Crosta et al. [26]. In fact, this strategy prevents ischemic lesions and immobilization syndromes but increases the risk of fatal intracerebral hemorrhages.

## Conclusion

Despite the lack of histopathological confirmation, our paper confirmed the previous investigations about CAA-ri, evidencing that this inflammatory condition has often a poor prognosis with multiple relapses and autoimmune, pulmonary and thrombotic complications too. Due to the progressive clinical worsening and the controversial therapeutic management, is fundamental a timely and proper clinico-radiological evaluation in suspected CAA-ri cases. Additionally, research is also needed to determine the role of CSF-Aβ autoantibodies. As biomarkers, they could support diagnosis of ARIA and CAA-ri and contribute to a better risk assessment of steroid and/or immunosuppressant administration in these two interrelated and maybe overlapped conditions.

## Ethical approval

Ethical approval was obtained from our institutional ethics committee.

## Funding

The author(s) received no financial support for the research, authorship and/or publication of this article.

## Informed consent

Written informed consent was retrospectively obtained from a legally authorized representative for anonymized patient information to be published in this article.

## Declaration of Competing Interest

The authors declared no potential conflicts of interest with respect to the research, authorship and/or publication of this article.
